# Genotyping validates the efficacy of photographic identification in a capture‐mark‐recapture study based on the head scale patterns of the prairie lizard (*Sceloporus consobrinus*)

**DOI:** 10.1002/ece3.7031

**Published:** 2020-11-18

**Authors:** Sarah A. Tomke, Chris J. Kellner

**Affiliations:** ^1^ Department of Forestry & Natural Resources University of Kentucky Lexington KY USA; ^2^ Department of Biology Arkansas Tech University Russellville AR USA

**Keywords:** capture‐mark‐recapture, double‐marking, genetic fingerprinting, genotyping, I^3^S, individual recognition, lizard, photographic identification

## Abstract

Population studies often incorporate capture‐mark‐recapture (CMR) techniques to gather information on long‐term biological and demographic characteristics. A fundamental requirement for CMR studies is that an individual must be uniquely and permanently marked to ensure reliable reidentification throughout its lifespan. Photographic identification involving automated photographic identification software has become a popular and efficient noninvasive method for identifying individuals based on natural markings. However, few studies have (a) robustly assessed the performance of automated programs by using a double‐marking system or (b) determined their efficacy for long‐term studies by incorporating multi‐year data. Here, we evaluated the performance of the program Interactive Individual Identification System (I^3^S) by cross‐validating photographic identifications based on the head scale pattern of the prairie lizard (*Sceloporus consobrinus*) with individual microsatellite genotyping (*N* = 863). Further, we assessed the efficacy of the program to identify individuals over time by comparing error rates between within‐year and between‐year recaptures. Recaptured lizards were correctly identified by I^3^S in 94.1% of cases. We estimated a false rejection rate (FRR) of 5.9% and a false acceptance rate (FAR) of 0%. By using I^3^S, we correctly identified 97.8% of within‐year recaptures (FRR = 2.2%; FAR = 0%) and 91.1% of between‐year recaptures (FRR = 8.9%; FAR = 0%). Misidentifications were primarily due to poor photograph quality (*N* = 4). However, two misidentifications were caused by indistinct scale configuration due to scale damage (*N* = 1) and ontogenetic changes in head scalation between capture events (*N* = 1). We conclude that automated photographic identification based on head scale patterns is a reliable and accurate method for identifying individuals over time. Because many lizard or reptilian species possess variable head squamation, this method has potential for successful application in many species.

## Graphical abstract

Photographic identification involving automated photographic identification software has become a popular and efficient noninvasive method for identifying individuals based on natural markings. We evaluated the performance of the program Interactive Individual Identification System (I^3^S) by cross‐validating photographic identifications based on the head scale pattern of the prairie lizard (*Sceloporus consobrinus*) with individual microsatellite genotyping in a large (>800 lizards) multi‐year field‐based capture‐mark‐recapture study. Individual identification using this method was highly successful; recaptured lizards were correctly identified by I^3^S in 94.1% of cases with an estimated false rejection rate (FRR) of 5.9% and a false acceptance rate (FAR) of 0%.

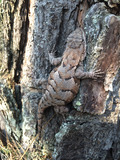

## 1 INTRODUCTION

Population studies often incorporate capture‐mark‐recapture (CMR) techniques to gather information on long‐term biological and demographic characteristics (Kacoliris et al., 2009, Sreekar et al. 2013). To achieve this, CMR approaches require all individuals to be uniquely marked so that they can be distinguished from conspecifics within a population. These markings must also be stable over time to ensure accurate reidentification (Arzoumanian et al., 2005).

Marking techniques for herpetofauna usually involve invasive methods such as tattooing (Clark, 1971; Hitchmough et al., 2012), attachment of color‐coded tags (Fisher & Muth, 1989; Galdino et al., 2014), inserting passive integrated transponder (PIT) tags (Camper & Dixon, 1988), or, most commonly, toe‐clipping (Kacoliris et al., 2009, Sacchi et al., 2010, Sreekar et al. 2013). However, applying these methods can elevate stress levels (Langkilde & Shine, 2006; Le Galliard et al., 2011), cause injury (Hitchmough et al., 2012; Klawinski et al., 1994), affect locomotion (e.g., climbing ability [Bloch & Irschick, 2005], running speed [Schmidt & Schwarzkopf, 2010]), and decrease survival (Camper & Dixon, 1988; Olivera‐Tlahuel et al., 2017). Furthermore, failure to accurately identify recaptured individuals due to loss of markers over time or natural toe loss can lead to biased estimates in population parameters, which could undermine research objectives (Drechsler et al., 2015; Hitchmough et al., 2012; Stevick et al., 2001).

Advances in molecular approaches have led to increasing use of genetic fingerprinting as an alternative identification tool (Taberlet & Luikart, 1999). Highly polymorphic molecular markers such as microsatellites or single nucleotide polymorphisms (SNPs) can be genotyped to provide a unique genetic combination that identifies each individual and can be used in a CMR framework (Drechsler et al., 2015; Lukacs & Burnham, 2005). Advances in laboratory techniques have enabled researchers to utilize this method in a noninvasive way by extracting DNA from shed tissues (Bauwens et al., 2018; Magoun et al., 2011; Piggott & Taylor, 2003), and is especially effective for long‐term studies since genetic markers do not change over time. Unfortunately, genetic sampling is costly, requires extensive laboratory processing, and is predisposed to genotyping errors, which can cause misidentification of individuals if not cross‐validated (Drechsler et al., 2015).

A more affordable and promising noninvasive technique uses photographic identification to identify individuals based on natural markers such as color or spot patterns (Bendik et al., 2013; Correia et al., 2014; Speed et al., 2007), scalation patterns (Bauwens et al., 2018; Dunbar et al., 2014; Kellner et al., 2017; Sacchi et al., 2010), or body contours (Gosselin et al., 2007; Markowitz et al., 2003). This method allows researchers to work on species that are difficult to capture or are threatened or endangered so that capture and handling are restricted (Dunbar et al., 2014; Moro & MacAulay, 2014). Photographs of individuals are stored in a digital database where they can be crossmatched by eye or by automated photographic identification software (e.g., Bolger et al., 2012; Matthé et al., 2008; Moya et al., 2015; van Tienhoven et al., 2007). The latter is faster and more accurate for large photographic databases (Drechsler et al., 2015).

Some studies have assessed the accuracy of automated photographic identification software (e.g., Dunbar et al., 2014; Kellner et al., 2017; Sannolo et al., 2016, Speed et al., 2007; Sreekar et al., 2013); however, few evaluations have employed a double‐marking system (e.g., Bendik et al., 2013; Drechsler et al., 2015; Sreekar et al., 2013). Cross‐validating photographic identification with a different technique yields more precise error rates because each method relies on different parameters (e.g., variable spot patterns, highly polymorphic microsatellites [Drechsler et al., 2015]), thus leading to different misidentifications and allowing for cross‐validation between the two methods. Further, it is unclear whether photographic identification is effective in multi‐year studies because in some species natural markings can change over time due to ontogenetic affects (Bendik et al., 2013; Germano & Williams, 2007; Treilibs et al., 2016) or damage to the skin (Bauwens et al., 2018; Moro & MacAulay, 2014). Those changes may reduce the ability for photographic identification programs to correctly identify recaptured individuals over time. To our knowledge, only Sacchi et al. (2010) compared misidentification rates for recapture events between years. However, their assessment was conducted by using a mock CMR trial from a known database and was not cross‐validated with an independent identification approach.

Here, we test the performance of the semiautomated photographic identification software Interactive Individual Identification System (I^3^S Classic; van Tienhoven et al., 2007), which has been used to identify lizards in population studies (e.g., Gardiner et al., 2014; Moro & MacAulay, 2014; Sreekar et al., 2013; Treilibs et al., 2016). Although usually applied to lizards that have variable coloration patterns (e.g., Moro & MacAulay, 2014; Treilibs et al., 2016; Sreekar et al., 2013), the program can be used to identify individuals based on scale patterns (e.g., Gardiner et al., 2014; Kellner et al., 2017; Sacchi et al., 2010). However, the accuracy of scale patterns as an identification marker has not been tested by using a double‐marking system. Therefore, we cross‐validated identifications that were based on scale patterns with genetic fingerprinting. We collected data in 2016 and 2017 and evaluated 863 captures of prairie lizards (*Sceloporus consobrinus*; hereafter prairie lizard). Our two objectives for this study are: (a) determine misidentification rates of I^3^S in a CMR environment by cross‐validating capture histories with genotyping, and (b) compare the relative ability of I^3^S to correctly identify recaptures made within the same year and between different years.

## 2 METHODS

### 2.1 Study species and study area

**FIGURE 1:**
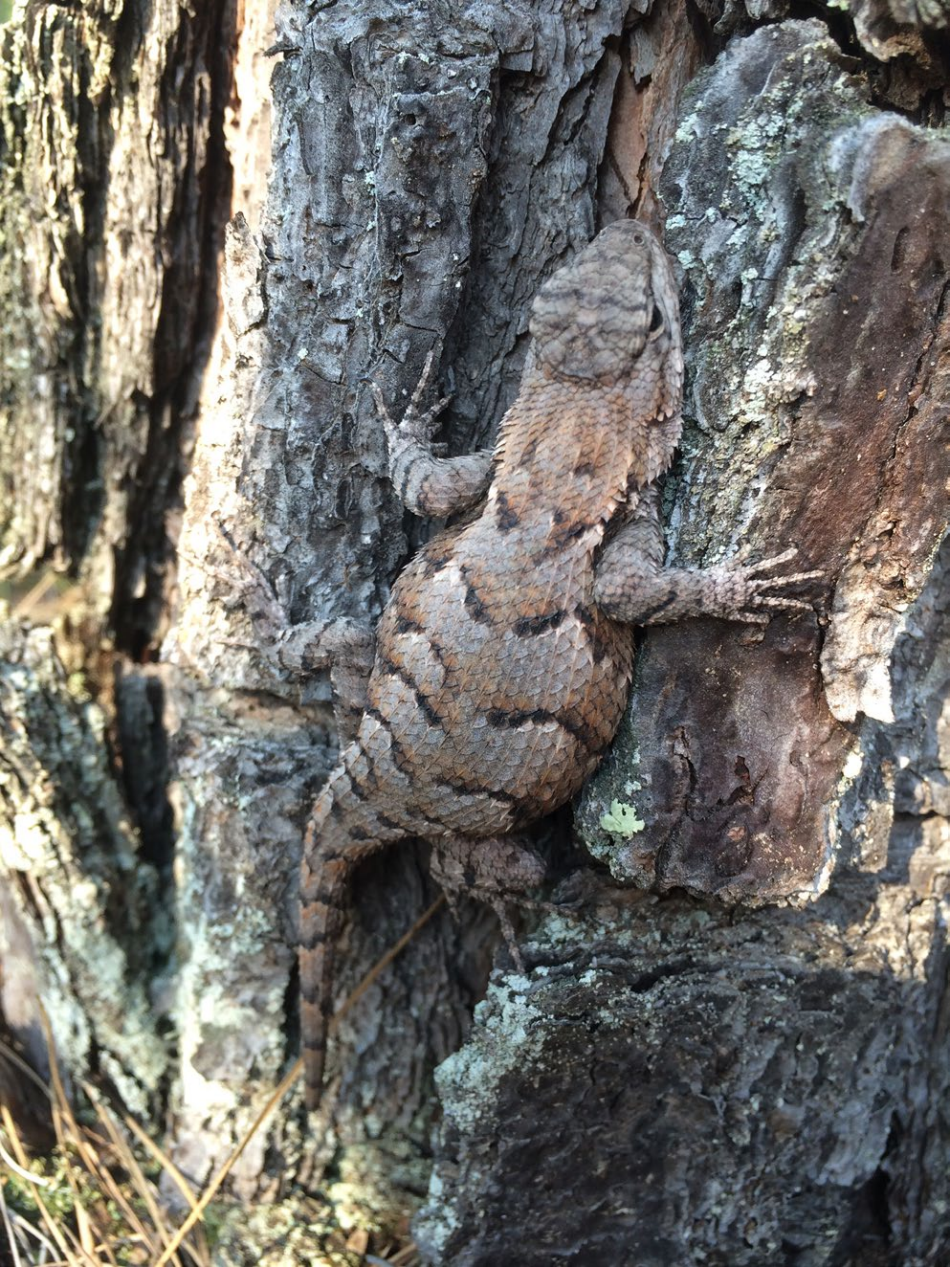
An adult female prairie lizard (*Sceloporus consobrinus*)

Prairie lizards are small phrynosomatid lizards (<70 mm from snout to vent; Smith et al., 1992; Figure 1). Their range spans from New Mexico to the Mississippi River and from northern Nebraska to central Texas (Leaché, 2009). Prairie lizards are primarily considered a forest edge species but also inhabit open areas (Conant & Collins, 1998). We collected lizards within 30 kilometers of Russellville, Arkansas, USA. Russellville is located in southwestern Pope County northeast of the Arkansas River. The city lies within the Arkansas River Valley between the Ozark and Ouachita National Forests. We captured lizards at 22 sites, which included anthropogenic rock piles along the Arkansas River, Lake Dardanelle, and Fourche Le Fave River, and forested trails at local county and state parks. Distance between sites ranged from 67 km to 133 m (albeit separated by the Illinois Bayou). Most sites were isolated from each other by highways or bodies of water and migration was presumed possible, but unlikely, for some closely located sites separated by suboptimal habitat. Prairie lizards were captured by noose from April to September of 2016 (*N* = 423) and 2017 (*N* = 440).

### 2.2 Photographic identification

We took close‐up images of the dorsal head scales of 863 captured lizards by using a Canon EOS Rebel T3 Digital SLR Camera and EF‐S 18–55 mm f/3.5–5.6 IS lens attached to a 36 mm extension tube. The camera was inserted into a wooden stand and lizards were held against the base of the stand at a 90° angle to the lens. This ensured a relatively consistent orientation across images. Even lighting was provided by an Aputure Imaging Industries, Amaran Halo ring flash (model number HC100; see Kellner et al., 2017 for more details). The images were combined into one digital database and processed within the computer software program Interactive Individual Identification System (I^3^S Classic ver. 4.0; van Tienhoven et al., 2007) following the methods described by Kellner et al. (2017) to create a 2D “fingerprint” for every individual based on dorsal head scale intersections. Briefly, three points including the anterior center of the rostral scale and the lateral most corners of the parietal scales were marked as reference points in each image. Up to 30 scale intersections encompassing the parietal, frontoparietal, frontal, and prefrontal scales were manually marked, which created a “fingerprint” unique to the image. To identify potential matches, I^3^S compares the fingerprint of a lizard to every other fingerprint in the database and lists the 50 closest matches in descending order. Each pairwise comparison is given a similarity score, which is based on the summed distance between matched pairs of points (i.e., matched scale intersections for two images). Thus, a low similarity score indicates that two images represent lizards that have similar scale patterns. The user then determines whether the image represents a recaptured or new individual by visually examining the pair of photos. This program also allows the user to name each image and assign a sex (e.g., “male,” “female,” or “unknown”) during the initial processing, which constrains the search to specific criteria. We gave each image a unique name that incorporated the site where the lizard was captured. We also assigned the image a sex when known. These additional data helped to narrow down potential matches during visual examination. To ensure consistency between years, the same researchers processed all the images in I^3^S.

### 2.3 Genotyping and genetic identification

We obtained genetic material for genotyping by collecting blood samples from the post‐orbital sinus of each lizard (MacLean et al., 1973). Each sample was immediately placed in 75% ethanol and stored at −20°C. Genomic DNA was extracted by using a FastID DNA extraction kit (Genetic ID NA, Inc.) and stored in 1× TE buffer solution. We amplified 11 microsatellite loci characterized for the eastern fence lizard (*Sceloporus undulatus*; Scun3, Scun7, Scun10, Scun11, Scun14, Scun15, Scun16, Scun19, Scun21, Scun22, and Scun23 [Lance et al., 2009]). Microsatellite loci were amplified separately in a 12 µl reaction containing 50 ng of genomic DNA, One*Taq* Hot Start 2X Master Mix (New England Biolabs), 10 µM fluorescently labeled primers, and 30 v/v % BSA. General PCR reactions consisted of one cycle of denaturing at 95°C for 2 min; 30–40 amplification cycles; and a 5 min 65°C final extension. Amplification cycle parameters varied per locus to incorporate the variable melting temperatures among loci. For Scun10 and Scun16, amplification cycle parameters involved 40 total cycles for 30 s each of: 96°C denaturation; 20 touchdown cycles (annealing: 65–55°C, decreasing 0.5°C per cycle) then 20 cycles at 55°C; and 72°C extension. For all other loci, amplification cycle parameters involved 30 total cycles of: 95°C denaturation for 30 s; 10 touchdown cycles for 45 s (annealing: 55–45°C, decreasing 1°C per cycle) then 20 cycles at 45°C for 45 s; and 1 min 68°C extension. Final PCR products were sent to the University of Missouri DNA Core Facility (Columbia, Missouri) for fragment analysis. We manually genotyped all loci by visually assessing electropherograms in Geneious version 11.0.3 (Kearse et al., 2012). Reamplification was performed for samples missing allelic data due to PCR or fragment analysis failures, or samples in which allelic signals were ambiguous.

To determine the likelihood that two lizards within each sample site shared a genotype, we calculated the probability of identity (PI) that two distinct individuals (a) shared identical genotypes, (b) differed at one locus, and (c) differed at two loci in GenAlEx 6.5 (Peakall & Smouse, 2012). The PI is the probability of two different individuals sharing the same genotype by chance alone; therefore, a high PI would indicate that two samples sharing the same genotype probably came from two distinct individuals, whereas a low PI would indicate that two samples sharing the same genotype probably came from one individual. We used the program GENECAP (Wilberg & Dreher, 2004) to identify individuals in the dataset that had identical genotypes, and genotypes differing by one or two alleles. Slight allelic variations between otherwise identical genotypes can be caused by PCR or genotyping errors. Therefore, in cases where individual genotypes differed by one or two alleles, we visually reassessed electropherogram files to determine if the observed variability was correct or due to error.

### 2.4 Performance of identification methods

To assess the performance of I^3^S, we calculated two metrics similar to false‐positive and false‐negative error rates that are commonly used in biometric performance assessments: false acceptance rate (FAR) and false rejection rate (FRR [Jain, 2007]). We defined FAR as the frequency of falsely identifying two distinct individuals as recaptures in I^3^S:

$$\text{FAR}=\frac{\#\text{false}\;\text{acceptances}}{\#\text{potential}\;\text{false}\;\text{acceptances}}$$

We defined FRR as the frequency of failing to correctly match two individuals as recaptures in I^3^S:

$$\text{FRR}=\frac{\#\text{false}\;\text{rejections}}{\#\text{true}\;\text{recaptures}}$$

To evaluate these metrics in our dataset, we used a similar approach as described by Bendik et al. (2013) in which capture histories for two independent identification methods were compared manually to determine where misidentification errors occurred. We considered successfully identified recaptures as individuals identified as a match in I^3^S and possessed identical genotypes indicated by GENECAP. Falsely accepted recaptures were individuals matched in I^3^S but had different genotypes. As mentioned above, genotyping errors can cause small, but false, allelic variations between genotypes of the same individual. Thus, the number of falsely accepted recaptures can be artificially inflated if small discrepancies among genotypes are not re‐evaluated. To prevent this, we visually assessed the electropherograms in tandem with photographs of the matched individuals from I^3^S. When scale patterns of the matched images were confirmed to be identical, we concluded that the observed variation in genotypes was due to genotyping errors and considered the I^3^S identification a successfully identified recapture. Falsely rejected recaptures did not have a match in I^3^S but shared an identical genotype with another sample. As before, electropherograms and photographs of the paired individuals were visually assessed in tandem to determine if the nonmatch was a false rejection in I^3^S or a false acceptance in GENECAP. We applied the same procedure to identify individuals that were potentially falsely rejected by both methods (i.e., paired individuals that did not have a match in I^3^S and had genotypes differing by only one or two alleles caused by genotyping error). When scale patterns of the paired individuals were determined to be identical, these individuals were considered false rejections by I^3^S.

### 2.5 Proficiency of photographic identification over time

To assess whether I^3^S could correctly identify recaptures of prairie lizards over time, we compared similarity scores and rankings of matched photographs between recaptures identified within the same year to those identified between different years. Further, we calculated the proportion of successfully identified recaptures, the FRR, and the FAR for each group by comparing recapture histories between I^3^S and genotyping.

## 3 RESULTS

### 3.1 Photographic identification analysis

**FIGURE 2:**
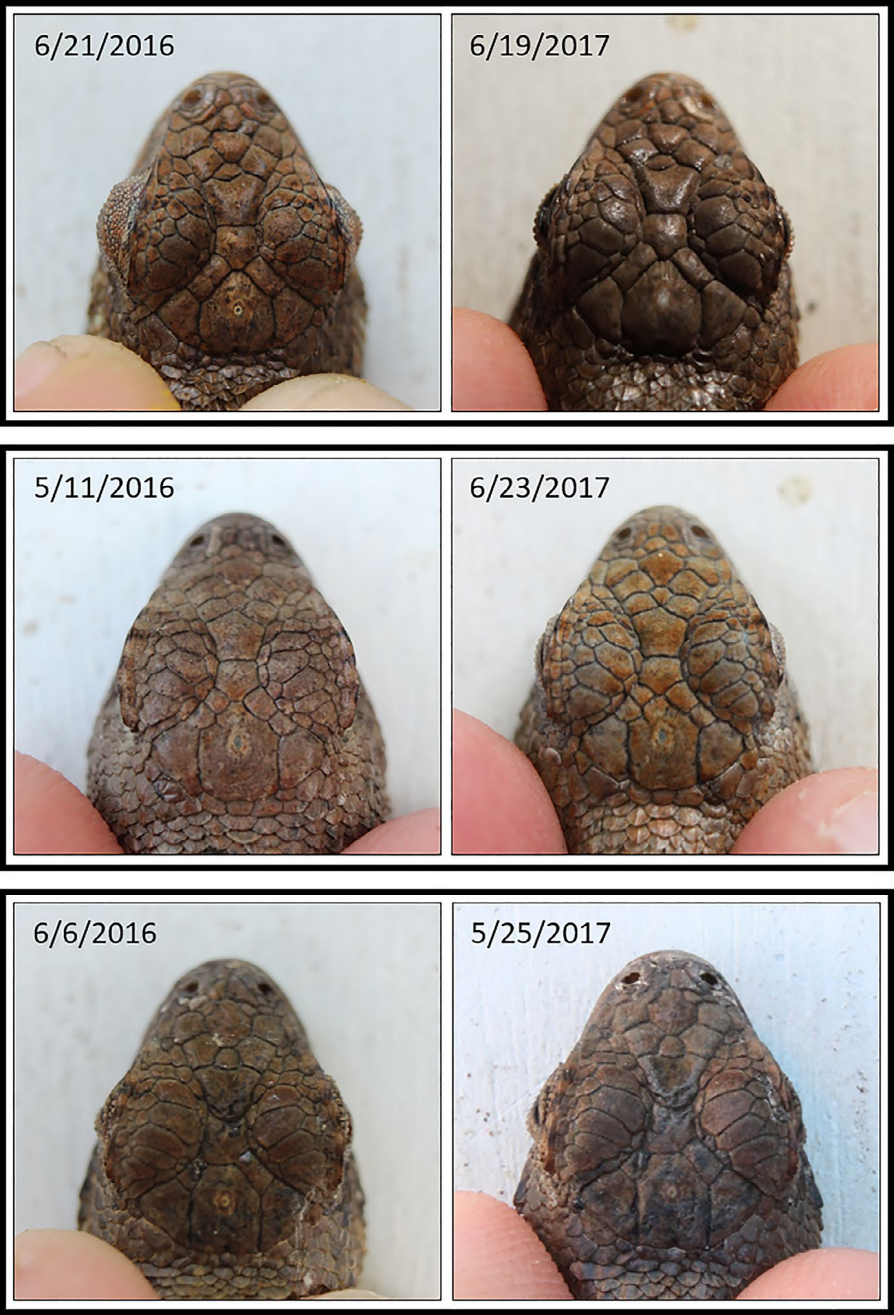
Examples of the stability of head scalation patterns in three lizards originally caught in 2016 and recaptured in 2017. Dates labeled on each image indicate date of capture

The I^3^S image database held 863 photographs of lizards captured in April‐September of 2016 and 2017. Of these, 161 were identified as recaptures by I^3^S independently of genotyping. Of the recaptures, 107 individuals were recaptured once, 22 were recaptured twice, two were recaptured three times, and one was recaptured four times. I^3^S returned the correct individual as the first matched image in 94.4% of searches and within the top ten matches in 99.4% of searches. Only one recapture (0.6%) was matched to an image ranked outside of the top 10; this individual was correctly paired with the 11th image. The I^3^S similarity scores for all recaptures ranged from 0.54 to 3.58 (median = 1.21; Q1 = 0.90; Q3 = 1.61). Overall, we found that in most cases scale patterns remained very stable between capture events (Figure 2).

### 3.2 Genetic identification analysis

We collected blood samples from 681 lizards in April‐September of 2016 and 2017 across 22 sites. Among sites, the average number of alleles per locus ranged from 4.6 to 11.5 ($\bar{X}$ = 8.7). Within sample sites, expected heterozygosity ranged from 0.665 to 0.824 ($\bar{X}$ = 0.776), observed heterozygosity ranged from 0.636 to 0.771 ($\bar{X}$ = 0.704), and the inbreeding coefficient (*F*_IS_) ranged from 0.033 to 0.245 ($\bar{X}$ = 0.123).

Of the 681 blood samples collected, GENECAP identified 103 individuals with matching genotypes: 98 shared identical genotypes, three differed by one allele, and two differed by two alleles. GENECAP did not find any genotypes that differed by three alleles. Data were missing for nine loci (0.42%) due to PCR or fragment analysis failures. The PI for individuals within sample sites that shared identical genotypes, that had genotypes differing at one locus, or that had genotypes differing at two loci were as follows: 3.0 × 10^–16^ to 1.1 × 10^–10^ ($\bar{X}$ = 5.9 × 10^–12^), 7.9 × 10^–15^ to 1.8 × 10^–9^ ($\bar{X}$ = 8.9 × 10^–11^), and 1.0 × 10^–12^ to 2.9 × 10^–8^ ($\bar{X}$ = 1.5 × 10^–9^), respectively. These results suggest DNA samples having identical genotypes or genotypes differing by one or two alleles likely came from the same individual.

### 3.3 Performance of I^3^S

The 103 individual pairs identified as potential recaptures in GENECAP also had photographs in the I^3^S database and were, therefore, used as our dataset to test the performance of I^3^S against genotyping. Of these, 96 (93.2%) individuals were identified as recaptures by both methods. Among the 96 recaptured pairs, five had mismatching genotypes at one allele. Visual assessment of photographs and electropherograms for these individuals indicated that all five genotypic mismatches were attributable to genotyping errors and the recaptures were considered successful identifications by I^3^S. Therefore, no false acceptances occurred by I^3^S.

**FIGURE 3:**
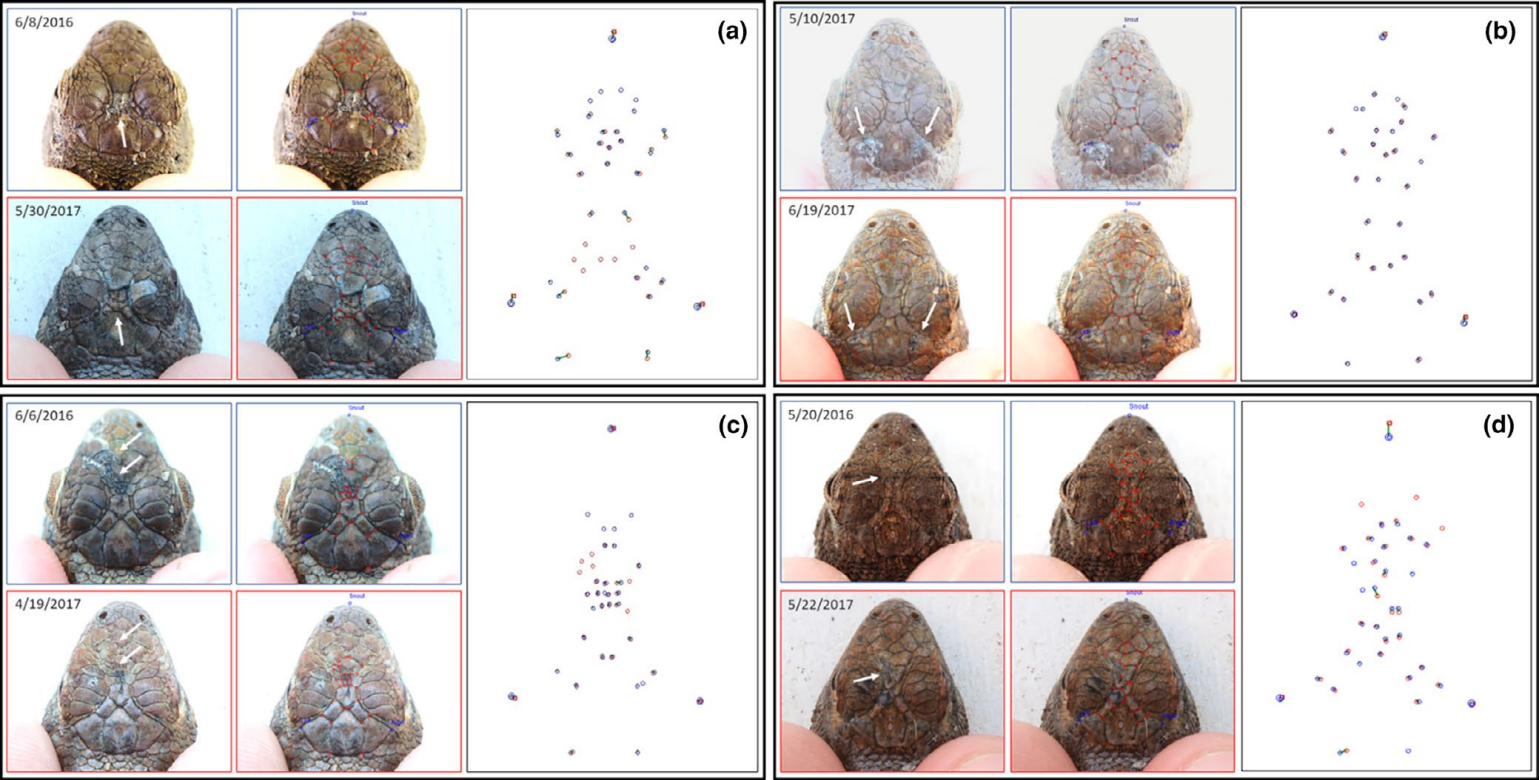
Examples of scale damage observed in individuals. Cases included one recaptured individual that was misidentified by I^3^S due to significant changes in fingerprints (a) and individuals where scale damage did not hinder their correct identification as a recapture (b–d). Each block consists of original photographs indicating the location of scale damage (white arrows), the scale intersections selected by the researchers in I^3^S to be incorporated into the fingerprint, and the overlapping fingerprints of the original and recaptured photograph created by I^3^S. Dates labeled on each image are the date of capture

**FIGURE 4:**
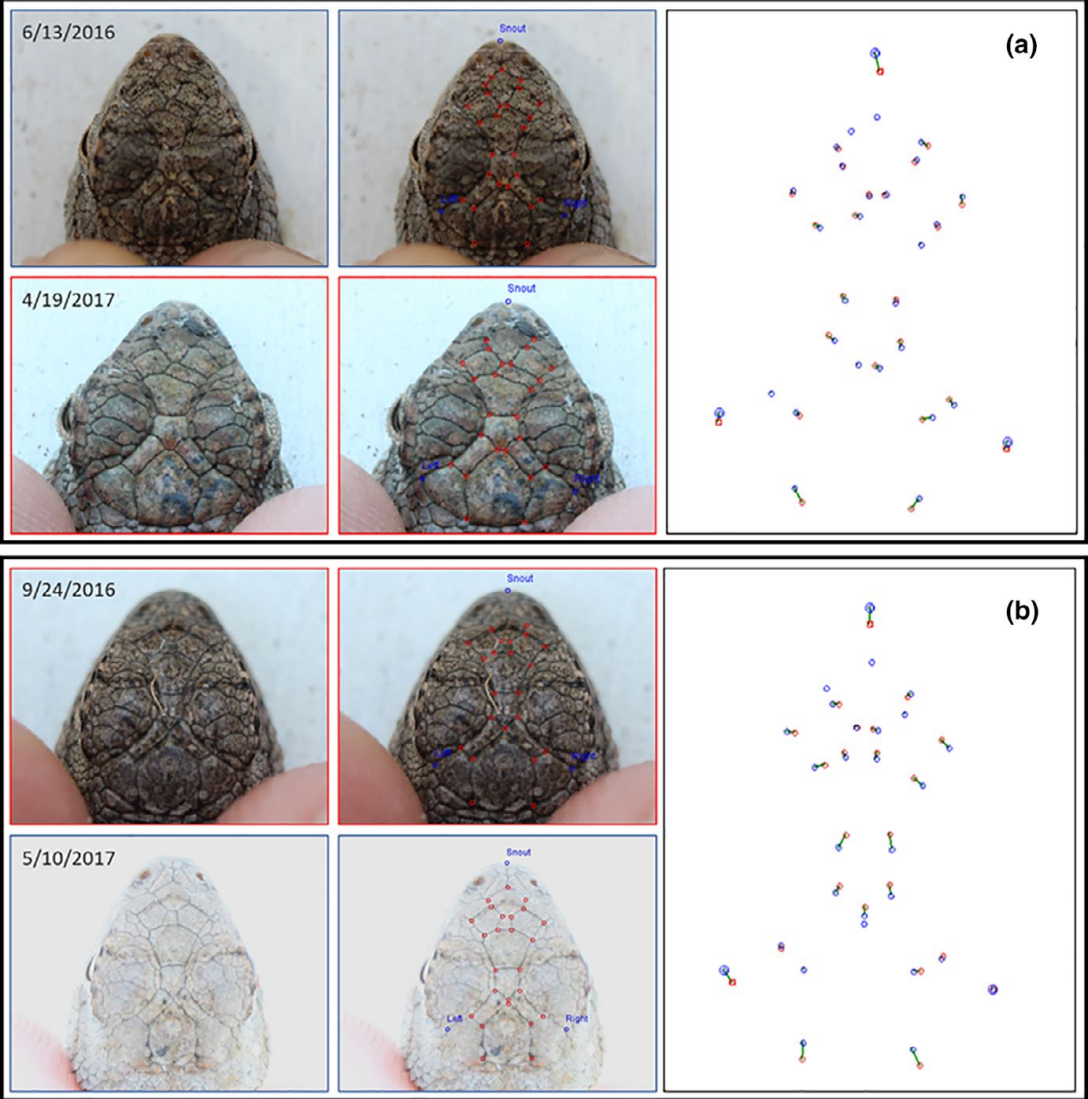
Examples of ontogenetic changes in crypsis and lepidosis observed in individuals that were originally captured as juveniles and recaptured as adults. Cases included changes in lepidosis that did not hinder the correct identification of recaptures by I^3^S (a) and one recaptured individual that was misidentified by I^3^S due to large allometric changes in scalation and subsequently identified through genotyping (b). Each block consists of original photographs, the scale intersections selected by the researchers in I^3^S to be incorporated into the fingerprint, and the overlapping fingerprints of the original and recaptured photograph created by I^3^S. Dates labeled on each image are the date of capture

Seven potential recaptured individuals were detected in GENECAP but were not detected by I^3^S: two pairs of individuals had identical genotypes, three pairs differed by one allele, and two pairs differed by two alleles. Visual assessment of photographs from individuals that had identical genotypes or that differed by one allele indicated that they were true recaptures. Photographs of individuals that had genotypes that differed by two alleles indicated one match as a true recapture and one match as different individuals. Therefore, I^3^S falsely rejected six recaptures. Since one genotype pair was identified as distinct individuals, our recapture sample size decreased to 102. In summary, I^3^S correctly identified 96 of 102 recaptured individuals (94.1%), falsely rejected six individuals (FRR = 5.9%) and did not falsely accept any individuals (FAR = 0%). Of the misidentified lizards, four were due to poor photograph quality, one was caused by scale damage within the fingerprint region of the head (Figure 3), and one was due to ontogenetic changes in lepidosis (e.g., the lizard was a young juvenile when it was first captured and an adult when it was recaptured; Figure 4).

### 3.4 Proficiency of I^3^S recapture identification over time

As indicated above, 161 recaptures were identified by I^3^S independently of genotyping from within our 863‐image database. Of these, 104 occurred within the same year and 57 occurred between 2016 and 2017. For recaptures within the same year, correct identifications were matched to the first ranked image in 101 cases (97.1%) and between the 2nd and 10th ranked images in three cases (2.9%). For recaptures occurring between years, correct identifications were matched to the first ranked image in 51 of 57 cases (89.5%), between the 2nd and 10th ranked image in five cases (8.8%), and the 11th ranked image in one case (1.7%). Although proportionally fewer recaptures were matched to the first image for between‐year recaptures than within‐year recaptures, the difference in rank distribution between the two groups was not significant (χ^2^ = 4.621, *df* = 2, *p* = .099). Similarly, median similarity scores were higher within (1.04) than between years (1.47). This difference was statistically different (W = 4,256, *p* ≤ .001).

When I^3^S and genotyping were combined, 102 recaptures were identified. Forty‐six of these were caught within the same year and 56 were caught between 2016 and 2017. As stated above, I^3^S falsely rejected six recaptures; one of which was caught within the same year and five were caught between years. The false rejection caught within the same year was misidentified due to poor photograph quality. Thus, I^3^S correctly identified 97.8% of recaptured individuals within the same year with a FRR of 2.2%. For lizards recaptured between years, I^3^S correctly identified 91.1% of recaptured individuals with a FRR of 8.9%. Three misidentifications were caused by poor photograph quality, one was caused by scale damage between capture events, and one was caused by ontogenetic changes in the scalation pattern. No false acceptances were identified in either group.

## 4 DISCUSSION

The intrinsic variability of pattern designs within wildlife populations makes natural markings an excellent alternative to traditional invasive marking techniques for CMR studies. Automated photographic‐recognition software is a potentially effective tool for identifying individuals based on natural markings within large datasets or when patterns are too complex for manual comparison (Bolger et al., 2012; Drechsler et al., 2015). However, few studies have assessed the accuracy of such programs by cross‐validating results with an independent identification approach.

Our findings demonstrate that the semiautomated photographic identification software I^3^S is a reliable tool for identifying individual prairie lizards based on head scale patterns. Our misidentification rate of 5.9% was lower than most error rates reported for I^3^S when used to identify individuals based on spot or line patterns (e.g., 8.4% for perenties (*Varanus giganteus*) [Moro & MacAulay, 2014] and 12% for male gliding lizards (*Draco dussumieri*) [Sreekar et al., 2013]) and was better than other studies in which scale patterns were used to distinguish individuals (e.g., 15.4% for hawksbill sea turtles (*Eretmochelys imbricata*) [Dunbar et al., 2014]; but see Sacchi et al., 2010).

Our results also indicate I^3^S outperforms other marking techniques used to identify lizards, including the genetic fingerprinting approach used in this study. Genotyping errors were present in nine of the 102 recaptured pairs identified when I^3^S and genetic fingerprinting were combined. These errors were only substantiated because photographic comparison of the paired individuals confirmed they were identical. Without this additional information, these recaptures would not have been identified, and the error rate based on genotyping alone would be 8.8%. Further, our I^3^S error rate was comparable or better than misidentification rates reported for toe‐clipping in other herpetofauna (Caorsi et al., 2012; Kenyon et al., 2009) and was not subjected to biases associated with natural toe loss, which has been documented in many lizards species (Bustard, 1971; Clarke, 1972).

Efficacy of I^3^S in our study was similar to efficacy reported in other multi‐year studies in which an automated photographic identification program was used to identify individuals. We found 2.2% and 8.9% of individuals were misidentified when recaptured within the same year and between years, respectively. However, when omitting misidentifications caused by poor photograph quality, these error rates decrease to 0% and 3.6% for within‐year and between‐year recaptures, respectively. In comparison, Sacchi et al. (2010) obtained a 2% error rate for recaptures occurring both within the same year and between two consecutive years in a blind mock CMR study. Further, they found that 3% fewer individuals recaptured between different field seasons were matched to one of the top five ranked images listed in the I^3^S output. Comparably, 2.5% fewer between‐year recaptures were matched to one of the top five ranked images in our study. These between‐year differences may be due to ontogenetic changes in scalation over time. This is further evidenced by the greater median similarity scores we observed for between‐year recaptures than within‐year recaptures. The change in median similarity score suggests that the relative positions of scale intersections changed slightly over time, that is, perhaps the scales are growing allometrically. Nevertheless, within our study that change did not impede our ability to identify individuals. Indeed, we found that adult squamation remained relatively stable between years (Figure 2), suggesting that this identification method would be reliable for CMR studies extending beyond two years. However, we do not know what effect allometric scale growth would have on a long‐lived species, or species with an extended juvenile development stage in its life‐history.

We found that six individuals (5.9%) were falsely rejected as recaptures, that is, the recapture was not correctly identified by I^3^S. Visual examination of the paired photographs for these individuals revealed most of the misidentifications (*N* = 4) involved blurry or underexposed images, which caused inaccurate identification of scale intersections by the user. This, along with variation in the angle of the subject with respect to the camera lens, is consistently reported to be the major contributor toward misidentification errors within manual and automated photographic identification systems (Correia et al., 2014; Stevick et al., 2001; Treilibs et al., 2016). The remaining misidentifications observed in this study were due to scale damage (*N* = 1; Figure 3a) and ontogenetic changes in lepidosis as one individual grew from a young juvenile to an adult (*N* = 1; Figure 4b); both of which caused significant differences between the I^3^S fingerprints constructed from the original and recaptured photographs.

Overall, these irregularities were uncommon, however, since the scale patterns of individuals were largely unchanged between capture events (Figure 2). Among the 167 recaptured individuals identified when I^3^S and genotyping were used independently, 15 (9.0%) had scale damage present in either the original or recapture photograph (Figure 3). This included wrinkled, scared, or missing scales. Among these, the individual was correctly paired with the first ranked image in I^3^S in 14 cases, indicating any changes in fingerprints between capture photographs were not significant enough to affect the efficacy of the program. Further, scale damage was observed in all regions of the head (e.g., parietal, frontal, and prefrontal), suggesting the location in which scale damage occurs does not influence the program's ability to identify individuals. Scale damage caused only one recapture to be misidentified (0.6%). The extent of that individual's scale damage was extreme, involving numerous adjacent frontal and parietal scales in the original photograph, which healed before it was recaptured one year later. Consequently, the I^3^S fingerprints were substantially different and the individual was falsely classified as a “new individual.”

Ontogenetic changes in lepidosis also had a very small effect on our ability to identify recaptured lizards (Figure 4). One individual, which was originally captured as a small juvenile and recaptured as an adult, was misidentified due to changes in head scale proportions (Figure 4b). In this example, enlargement of the parietal scales and elongation of the rostral region altered the I^3^S fingerprint developed for the recaptured image so significantly that the correctly paired image was not included in the list of 50 closest matches provided by the program. Consequently, the individual was only properly identified through genotyping. This result differed from a second juvenile captured in this study, which was correctly matched to the first paired image in I^3^S despite also being recaptured as an adult (Figure 4a). Consequently, we do not know whether ontogenetic changes in lepidosis would hinder the program's ability to identify recaptured lizards originally caught during very young age classes. Indeed, ontogenetic changes in scale patterns have been documented in numerous lizard species (Bruner et al., 2005; Lazić et al., 2017; Piras et al., 2011), and in some species the allometry of the scales (i.e., differences in growth rates among different scales) vary significantly among individuals (Bruner et al., 2005; Lazić et al., 2017). Further, cryptic coloration and underdeveloped scales on the medial region of the heads of hatchlings prevented the construction of fingerprints in I^3^S for that age class. However, we noticed that scale margins on the supraoculars were quite distinct and could have been used to fingerprint hatchlings. Hatchlings lost the cryptic patterning and scale margins became well defined within the medial region of the head during the early juvenile stage; thus, these ontogenetic effects did not hinder the formation of fingerprints for juveniles, subadults, or adult lizards. Ontogenetic changes in coloration patterns have been documented for other lizard species as well (Burton, 2004; Treilibs et al., 2016), but its effect on the efficacy of photographic identification is small.

### 4.1 Application to other species

The software program I^3^S is an effective method for identifying individuals of many lizard species based on a variety of natural patterns or markings (Gardiner et al., 2014; Moro & MacAulay, 2014; Sacchi et al., 2010; Treilibs et al., 2016). Until recently, the application of I^3^S to lepidosis was only applied to pectoral and auricular regions of lizards that have highly variable scalation in those areas of the body (e.g., Gardiner et al., 2014; Sacchi et al., 2010; Strickland et al., 2014). Kellner et al. (2017) provided evidence that dorsal head scales are a valid alternative for lizards that have small and uniform scales in pectoral and facial regions. We believe this marking technique could be successfully applied in other lizard species that possess variable, relatively large, and asymmetric dorsal head scales. For example, these characteristics have been found in other phrynosomatid lizards (e.g., Fox, 1975; Wiens & Penkrot, 2002), iguanas (e.g., *Cyclura* sp. [Burton, 2004]), liolaemids (e.g., Lobo et al., 2007; Valladares et al., 2002), lacertids (e.g., Elbing & Rykena, 1996; Lue & Lin, 2008; Sánchez‐Vialas et al., 2019), and whorltail lizards (e.g., *Stenocercerus* spp. [Cadle, 1991, Venegas et al., 2016]). The technique could potentially be used on threatened or endangered species where harmful or unfavorable invasive techniques should be avoided (e.g., in the United States: reticulate collared lizard (*Crotaphytus reticulatus*), dunes sagebrush lizard (*Sceloporus arenicolus*), little white whiptail (*Aspidoscelis gypsi*), and sandstone night lizard (*Xantusia gracilis*). Further, our methodology could easily be applied to other reptile groups since many turtle and snake species possess individually distinct and variable head scales (Bauwens et al., 2018; Calmanovici et al., 2018; Dunbar et al., 2014).

## 5 CONCLUSION

The results of our double‐marking study indicate that I^3^S can accurately identify recaptured prairie lizard individuals based on head scalation patterns. All errors were attributed to falsely rejected recaptures. Most of these misidentifications were due to poor photograph quality. Two false rejections were caused by changes in head scale patterns due to scale damage and ontogenetic allometry. These anomalies were rare and should not deter the use of photographic identification based on head scalation because identification was successful for almost all recaptures that exhibited changes in scale patterns from injuries and growth. The use of this program should be explored with other lizard or reptilian species that possess variable dorsal head squamation as we believe this method has potential for successful application in many species.

## CONFLICT OF INTEREST

None declared.

## AUTHOR CONTRIBUTION

**Sarah Ann Tomke:** Conceptualization (equal); Data curation (lead); Formal analysis (lead); Funding acquisition (supporting); Investigation (lead); Methodology (lead); Project administration (equal); Resources (equal); Software (equal); Supervision (equal); Validation (equal); Visualization (lead); Writing‐original draft (lead); Writing‐review & editing (equal). **Chris J. Kellner:** Conceptualization (equal); Data curation (supporting); Formal analysis (supporting); Funding acquisition (lead); Investigation (supporting); Methodology (supporting); Project administration (equal); Resources (equal); Software (equal); Supervision (equal); Validation (equal); Visualization (supporting); Writing‐original draft (supporting); Writing‐review & editing (equal).

## Data Availability

Sampling locations, morphological data, and microsatellite genotypes are available at Dryad https://doi.org/10.5061/dryad.1rn8pk0s3.
